# Sedative Powers of the Root of Yellow Jessamine

**Published:** 1855-05

**Authors:** C. H. Cleaveland

**Affiliations:** Cincinnati


From the Amer Med Gazette, N Y.

*Sedative Powers of the Root of Yellow Jesamine.* By DR. CLEAVELAND, of Cincinnati.

MR. EDITOR: For some time past the profession in the West and South have had their attention called to root of the *Geeseminum Semfuvirens* or Yellow Jessamine, as possessed of very wonderful and valuable remedial properties; even those of the most opposite and contradictory nature having been ascribed to it.

The history of the discovery of its power is said to have been as follows: A planter of Mississippi, whose name is not remembered, while laboring under a severe attack of billious fever, which had not yielded to the remedies used, sent a servant into his garden to procure a root and prepare an infusion of it for him to drink. The servant, by mistake, collected the root of the Yellow Jessamine, made an infusion of it, and gave it his master to drink. — Soon after swallowing some of it, the planter lost his muscular power, so as to be unable to move a limb or to raise his eyelids; while he could hear and feel and exercise his usual faculties as well as in health.

His friends became much alarmed at his great prostration, but after some hours he recovered his muscular powers, and was highly pleased to find himself free from fever He soon learned from his servant what plant it was from which he obtained the root, and trying its effects upon the people of his own plantation and those of his neighbors, he ascertained that he had a valuable remedy for fevers. The use of the article became known to a quack, who prepared from the Gelsimum a nostrum which he styled the "Electrical Febrifuge," which was simply the tincture of the root of the Gelsimum, prepared with dilute alcohol, and disguised with the essence of winter-green.

After a time the true character of this tincture became known to the profession, and at the present time it is being used very frequently by many of this part of the country, who however seem from their reports not to have a very clear idea of its action on the system, or the class of cases for which it is indicated.

I have tested it uncombined with any other medicine, until I am satisfied that it is one of the most valuable sedatives at present known to the profession—and peculiarly valuable, as it expends its force mainly upon those nerves that are distributed to the muscles of locomotion leaving entirely unaffected the intellectual faculties, and probably the great ganglionic system of nerves.

We have in the Aconite a valuable sedative to the nerves of general sense, or an anæsthetic to those nerves which, when infringed upon, cause the sensation of pain—a real pain-destroyer—so far as the superficial sensatory nerves are concerned. Hence its value as an external application in all painful conditions.

In the veratrum viride we have an excellent sedative to the muscular system, and to depress vascular excitement; but, while it depresses the vagus nerve, decreasing the frequency of the contractions of the heart, and deadening the sensibility of the lungs, and thus relieving them of a sense of want of breathing, it also sometimes deranges the gastric nerves, producing such an amount of nausea, and even vomiting, as to preclude the further use of the remedy It also not unfrequently induces such free cathasis as thus to greatly injure the patient if its use is continued.

These objections do not obtain against the Gelseminum, for while it proves a most powerful sedative to the nerves supplying the muscles of locomotion, as in the case of the planter who first took it, it never seems to produce the slightest effect upon the brain, or in the least degree to derange the stomach or bowels.

It does lessen the pulse and the frequency of respiration, and while it produces a wonderful relaxation of all the muscles, it relieves all sense of pain by acting upon the nerves of general sense in a manner similar to aconite.

Usually not more than thirty or forty minutes elapse after the agent is taken into the stomach before the sedative is experienced; and if the dose be not repeated, its influence will continue only for an hour or two. Many practitioners, however, seem not to be aware of this, and order doses, in my opinion, unnecessarily large, and repeat them only once in six or eight hours.

As yet the Gelseminum has been but little used, except in the form of a tincture prepared with dilute alcohol; but, as in the instance of its first trial, experience proves that the sedative property is fully soluble in water; and the experience of some in New Orleans, who made use of a tincture prepared with strong alcohol, would indicate that the root contained a dangerous resinoid substance, readily soluble in strong alcohol, but not soluble in dilute liquor or in water. Some deaths even occurred in that city, which were supposed to be produced by this poisonous resinoid principle.

The dose of the saturated tincture, with dilute alcohol, is from three to thirty drops; and I think it much better and safer to give small doses frequently repeated, than to risk a too large dose of a powerful sedative medicine.

I need not call the attention of your readers to the great variety of cases in which sedatives are indicated, and in which those that act as lobelia or veratrum do, on the stomach or bowels, are *contra*-indicated. Perhaps no other class of agents would be more frequently resorted to than these, did the profession feel sure they could command the agent which would produce the desired effect.

From sufficient personal experience, as well as from the observations of many physicians who have used this preparation, I am satisfied that, as a sedative to the nerves branching from the spinal cord, and going to the organs of locomotion, or the nerves of voluntary motion, and in a lesser degree the vagus or sympathetic branches of nerves that are distributed to the heart and the lungs, inducing a less powerful and less frequent pulse, and a more sluggish and feebler respiration, the Gelseminum will prove highly satisfactory to any who may give it a trial.

It has had ascribed to it tonic, alterative, anti-periodic, and phlogistic, anti-spasmodic, diaphoretic, diuretia, and perhaps many other anomalous and contradictory properties; but, like the laudations of so many foolish friends, so much has been said, that but little, if any, of these statements by many have been believed.

It may be possessed of other powers, but my most careful testing of the drug has revealed to me only its sedative powers.

Yours, &c.,

Cincinnati, February, 1855. C. H. CLEAVELAND.

